# Associations of cardiovascular risk factors, carotid intima-media thickness and manifest atherosclerotic vascular disease with carpal tunnel syndrome

**DOI:** 10.1186/1471-2474-12-80

**Published:** 2011-04-26

**Authors:** Rahman Shiri, Markku Heliövaara, Leena Moilanen, Jorma Viikari, Helena Liira, Eira Viikari-Juntura

**Affiliations:** 1 Centre of Expertise for Health and Work Ability, Finnish Institute of Occupational Health, Helsinki, Finland; 2 Department of Health and Functional Capacity, National Institute for Health and Welfare, Helsinki, Finland; 3 Department of Medicine, Kuopio University Hospital, and University of Kuopio, Kuopio, Finland; 4 Department of Medicine, University of Turku, and Turku University Hospital, Turku, Finland; 5 Unit of General Practice, Helsinki University Central Hospital, University of Helsinki, Helsinki, Finland; 6 Kirkkonummi Health Centre, Kirkkonummi, Finland

**Keywords:** Atherosclerosis, carotid artery, coronary artery disease, hypertension, obesity, smoking, wrist

## Abstract

**Background:**

The role of atherosclerosis in carpal tunnel syndrome (CTS) has not previously been addressed in population studies. The aim of this study was to investigate the associations of cardiovascular risk factors, carotid artery intima-media thickness (IMT), and clinical atherosclerotic diseases with CTS.

**Methods:**

In this cross sectional study, the target population consisted of subjects aged 30 or over who had participated in the national Finnish Health Survey in 2000-2001. Of the 7977 eligible subjects, 6254 (78.4%) were included in our study. Carotid IMT was measured in a sub-sample of subjects aged 45 to 74 (N = 1353).

**Results:**

Obesity (adjusted odds ratio (OR) 2.4, 95% confidence interval (CI) 1.1-5.4), high LDL cholesterol (OR 3.8, 95% CI 1.6-9.1 for >190 vs. <129 mg/dL), high triglycerides (OR 2.7, 95% CI 1.2-6.1 for >200 vs. <150 mg/dL), hypertension (OR 3.4, 95% CI 1.6-7.4) and cardiac arrhythmia (OR 10.2, 95% CI 2.7-38.4) were associated with CTS in subjects aged 30-44. In the age group of 60 years or over, coronary artery disease (OR 1.9, 95% CI 1.1-3.5), valvular heart disease (OR 2.3, 95% CI 1.0-5.0) and carotid IMT (1.4, 95% CI 0.9-2.1 for each 0.23 mm increase) were associated with CTS. Carotid IMT was associated with CTS only in subjects with hypertension or clinical atherosclerotic vascular disease, or in those who were exposed to physical workload factors.

**Conclusions:**

Our findings suggest an association between CTS and cardiovascular risk factors in young people, and carotid IMT and clinical atherosclerotic vascular disease in older people. CTS may either be a manifestation of atherosclerosis, or both conditions may share similar risk factors.

## Background

Carpal tunnel syndrome (CTS) is the most common nerve entrapment [1,2]. It can cause major work disability and incur considerable costs to society [3]. Epidemiological studies have reported a higher risk of CTS among women than among men [1,4-6]. It is common in the third trimester of pregnancy, and in cases of hypothyroidism [7,8] and renal disease [9]. Among men [4,5,10] and women [4,10], the age distribution of CTS is bimodal, with a peak between the ages of 50 and 59 and a second peak among those aged 70 or over.

Symptoms of CTS may appear when there is edema in the carpal tunnel, the volume of the contents of the tunnel is increased, the vulnerability of the nerve is increased, or the blood supply of the nerve is impaired. Endothelial damage may increase vascular permeability and cause edema in the carpal tunnel [11,12]. Increased interstitial fluid pressure in the carpal tunnel causes compression of the carpal tunnel contents, especially the median nerve. This may lead to poor blood circulation in the flexor synovial cells and the median nerve. Histological studies have shown edema, vascular sclerosis and fibrous tissue in the flexor tenosynovium [13,14].

Conditions that may increase the vulnerability of the median nerve include diabetes [7,8,15], renal disease [9], smoking [16-18] and the toxic effects of alcohol [19]. Impairment of vascular supply may increase the vulnerability of the nerve to mechanical loads, and prolonged tissue ischemia can lead to degeneration of the nerve and intraneural fibrosis [12,20]. Tenosynovitis of the finger flexor tendons is the most common cause for increases in the contents of the carpal tunnel. It occurs commonly in rheumatoid arthritis [21] and as a result of high physical workload exposure [22]. Mechanical stress may play a greater role among young rather than old people [6]. In contrast, ischemia caused by cardiovascular diseases or atherosclerosis may play a major role among elderly people.

Some evidence exists on the role of atherosclerosis risk factors such as obesity, smoking, LDL cholesterol and metabolic syndrome in CTS [16,17,23-27]. However, there is no evidence so far to support an association between atherosclerosis and CTS. In this study, we investigated the associations of atherosclerosis risk factors, carotid intima-media thickness (IMT), a surrogate marker of early atherosclerosis, and clinical atherosclerotic diseases with CTS. We hypothesized that atherosclerosis plays a larger role in the pathogenesis of CTS among older people rather than among younger individuals. The higher prevalence of CTS among elderly people - the second peak of the bimodal age distribution of CTS - may be due to atherosclerotic vascular disease. Moreover, we hypothesized that the presence of atherosclerotic disease increases the vulnerability of the median nerve to mechanical stress due to physical exposures.

## Methods

### Population

In the national health examination survey, Health 2000, the target population comprised men and women aged 30 or over residing in Finland between the fall of 2000 and the spring of 2001 [6]. To obtain a representative sample of the whole Finnish population, a two-stage stratified cluster sampling design was used and sample stratified according to the five university hospital regions, each containing roughly one million inhabitants. From each university hospital region, 16 health care districts were sampled as clusters.

The purpose of this survey was to provide up-to-date information on major public health problems in Finland, their causes and treatment, as well as functional capacity and work ability [28]. Information was gathered by means of interview and clinical health examination. At the comprehensive health examination, specially trained nurses carried out a symptom interview on cardiovascular and musculoskeletal complaints, and physicians performed a standardized physical examination, which included assessing the status of the upper extremity.

The original sample consisted of 8028 subjects aged 30 or over. Of these, 51 died before the interview, 6986 (87.6%) were interviewed, and 6354 (79.7%) participated in the health examinations. Subjects with missing CTS information (n = 100) were excluded, leaving 6254 (78.4%) subjects eligible for the analysis [6].

The Ethical Committee of Epidemiology and National Welfare of the Helsinki University Hospital District approved the Health 2000 survey on the 21^st ^of September 1999. All participants gave their informed consent.

### Outcome

The diagnosis of possible CTS was based on 1) possible or classic/probable Katz hand diagrams (pain or paraesthesia or decreased sensitivity present in the thumb or index or middle finger during the preceding seven days), plus 2) either a positive Tinel's test result, combined wrist flexion and carpal compression, decreased sensation in the median nerve distribution, or weakness of thumb abduction or wasting of the thenar eminence [29]. A probable case of CTS was defined as a classic/probable Katz hand diagram (symptoms in two of the three radial fingers), and positive findings in at least two of the four clinical tests. We also gathered information regarding surgery due to CTS.

### Determinants

#### Atherosclerosis risk factors

*Smoking *status was assessed by a home interview and the subjects were defined as 1) current smokers if they smoked cigarettes, cigars or a pipe at the time of the interview; 2) former smokers if they had smoked for at least one year in the past and were not current smokers; 3) occasional smokers; and 4) never smokers. For current smokers, pack years were estimated and grouped into three levels (<10, 10-20, >20). *Leisure-time physical activity *was assessed by a single global question; "How often do you exercise so that you are short of breath or sweating?" We classified physical activity into three levels: ≤ 1, 2-3, and ≥ 4 times per week.

Weekly *consumption of alcohol *was recorded in units (drinks, serving portions) and converted into grams of absolute alcohol. Alcohol consumption was grouped into 4 levels, none (0 grams of alcohol), light, moderate, or heavy (the three latter were based on tertiles of the distribution).

Height, weight, waist circumference and hip circumference were measured. *Body mass index *(BMI) was classified into underweight (BMI <18.5 kg/m^2^), normal weight (BMI 18.5-24.9 kg/m^2^), overweight (BMI 25-29.9 kg/m^2^) and obesity (BMI ≥30 kg/m^2^). *Waist circumference *was grouped into three levels; for men <94 cm, 94-101.9 cm, and ≥102 cm, and for women <80 cm, 80-87.9 cm and ≥88 cm. *Waist-to-hip ratio *was classified into three groups: for men <0.9, 0.9-1.0, and >1.0, and for women <0.8, 0.8-0.9, and >0.9.

The diagnosis of *hypertension *was based on a systolic blood pressure of ≥140 mm Hg or a diastolic blood pressure of ≥90 mm Hg, or a previous diagnosis of hypertension together with the use of blood pressure lowering medication. The diagnosis of *diabetes *was based on elevated fasting blood glucose, and/or a known previous diagnosis of diabetes, or glucose lowering medication.

Fasting blood samples were collected for the analysis of serum glucose, insulin, cholesterol, and triglycerides. We defined *metabolic syndrome *according to the criteria of the American Association of Clinical Endocrinologists [30], i.e. when at least three of the following criteria were present: 1) Central obesity, defined as waist circumference >102 cm for men and >88 cm for women; 2) high fasting triglycerides, defined as ≥151 mg/dl; 3) low high-density lipoprotein cholesterol defined as <40 mg/dl for men and <50 mg/dl for women; 4) elevated blood pressure, defined as a systolic blood pressure of ≥130 mm Hg or a diastolic blood pressure of ≥85 mm Hg; and 5) impaired fasting glucose, defined as a fasting glucose of ≥110 mg/dl. The homeostasis model assessment of *insulin resistance *was defined as serum insulin × glucose/22.5. High-sensitive serum *C-reactive protein *was defined as ≥ 3 mg/l.

#### Atherosclerotic diseases

Information on cardiovascular diseases was obtained through interview and clinical examination. The diagnosis of *coronary artery disease *was based on a physician ascertained diagnosis of previous angina pectoris, myocardial infarction, coronary angioplasty or a by-pass operation. The diagnosis of *cerebrovascular disease *was based on a history of previous stroke or a transient ischemic attack. Other heart or vascular diseases were *heart failure*, *cardiac arrhythmia*, *valvular disease*, or *intermittent claudication*, and the diagnoses were based on history.

#### Carotid artery intima-media thickness

Earlier [31] we described the details of ultrasound measures of carotid artery intima-media thickness (IMT). Ultrasound measures of carotid IMT were performed in a sub-sample of men and women aged 45 to 74 who resided within 200 kilometers of the six study clinics that had cardiovascular ultrasound equipment with a linear array transducer available. The six study clinics covered six Finnish towns and their surrounding areas (Helsinki, Turku, Tampere, Kuopio, Joensuu, and Oulu). Subjects (N = 1867) who fulfilled these eligibility criteria were invited and 1526 (82%) of them participated in the carotid artery ultrasound study. We assessed the relation between IMT and CTS among 1353 (72%) subjects whose data on both carotid artery ultrasound and CTS was available.

A high-resolution B-mode carotid ultrasound examination of the right carotid artery was performed first on the distal 1 cm of the common carotid artery and then on the carotid artery bulb. The IMT was measured from three digitized end diastole images of the common carotid artery (lateral angle) and the carotid bulb (three interrogation angles). We used an average of these six measures in the analysis of this study.

#### Covariates

The home interview elicited information on *age*, *gender*, *years of education*, and *work-related physical load factors*. The presence of the following physical exposures in the current job was elicited (frequency or duration per day); working with hands above the shoulder plane, manual handling of loads over 5 kg, manual handling of loads over 20 kg, working with a vibrating tool, work demanding high handgrip forces, and repetitive movements of the hands or wrists [6]. In our previous report [6] we showed that in the presence of all physical load factors in the model, only high handgrip forces for at least one hour and using vibrating tools for at least two hours were associated with CTS. Therefore in the current study we controlled the obtained odds ratios of CTS for *high handgrip forces *and *using vibrating tools*.

The presence of *somatization *was assessed using the 13-item somatization part of Symptom Check List-90 [32]. We excluded three questions on pain and scored the remaining 10 items on a five-point Likert scale. The total score for each subject ranged from 0 to 40, with higher scores reflecting higher levels of somatization. In our earlier report, we tested a range of psychological and psychosocial factors. In the presence of all these factors we found an association with somatisation only. We therefore controlled for only this factor.

### Statistical methods

The statistical significance level was set at P < 0.05. We adjusted P-values for multiple testing using Bonferroni correction and set statistical significance level at P-value ≤ 0.003 for 15 subgroup analyses. Logistic regression models were run to study the associations of atherosclerosis risk factors, carotid IMT, and clinical vascular diseases with CTS. We used three outcomes (possible and probable CTS combined, probable CTS, surgery due to CTS) to take into consideration the severity of the disorder and the likelihood of correct diagnosis. We performed survey data analysis by using Stata's *svy *prefix command. Survey data analysis considers the weighting, clustering, and stratification of the survey design to correct imbalances in the probabilities of selection and to estimate the right standard errors. Stata's default *svy *variance estimator, the linearized variance estimator, was used to compute standard errors [33]. In the nonsurvey data analysis, this variance estimator refers to as the robust variance estimator. Confidence intervals were calculated based on the number of observations in the specific group being analysed. Age (continuous), gender, years of education (continuous), somatization (continuous), high handgrip forces and using vibrating tools were included in the logistic regression models as confounders. Gender- and age-specific analyses were also carried out. Effect modification was studied using stratified analysis. Multiplicative interactions were tested by including product terms in the multivariable models between physical load factors, risk factors of atherosclerosis, carotid IMT (categorized into two groups using the median) and clinical atherosclerotic disease.

## Results

### Background characteristics

The mean age of the study population was 52 and 48% were men. One-fourth of the population was current smokers and the mean BMI was 26.2 kg/m^2 ^(Table 1). The most common cardiovascular diseases were hypertension and coronary artery disease. The mean carotid IMT was 0.93 mm (range 0.5-2.5 mm). The prevalence of possible and probable CTS combined was 3.8%, probable CTS 1.0%, and operated CTS 1.3%.

**Table 1:** Background characteristics of study subjects (weighted proportion or mean), Health 2000 Survey, 2000-2001.

Characteristic	Mean (SD)	% (95% CI)
Age (years)	51.9 (13.9)	
Years of education	11.3 (4.0)	
		
*Smoking status*		
Former		31 (30-32)
Current		26 (24-27)
		
*Weight-related*		
Body mass index (kg/m^2^)	26.2 (4.3)	
Waist circumference (cm)	93 (13.3)	
Hip circumference (cm)	102 (9.5)	
		
*Serum lipids*		
LDL cholesterol (mg/dL)	148 (46)	
HDL cholesterol (mg/dL)	52 (15)	
Total cholesterol (mg/dL)	232 (44)	
		
*Metabolic factors*		
Metabolic syndrome		30 (29-32)
Diabetes		5.2 (4.6-5.9)
		
*Hypertension*		20 (18-21)
		
*Cardiovascular diseases*		
Coronary artery disease		7.1 (6.4-7.8)
Heart failure		1.7 (1.3-2.0)
Arrhythmia		5.1 (4.5-5.7)
Valvular heart disease		2.5 (2.1-3.0)
Cerebrovascular disease		2.7 (2.3-3.1)
Intermittent claudication		1.2 (0.9-1.5)
		
*Atherosclerosis*		
Carotid intima-media thickness (mm)	0.93 (0.23)	
		
*Individual psychological factor*		
Somatization (score 0-40)	6.7 (5.8)	
		
*Carpal tunnel syndrome*		
Possible or probable		3.8 (3.3-4.4)
Probable		1.0 (0.7-1.3)
Operated		1.3 (1.0-1.6)

Men had higher BMI, waist circumference and carotid IMT and lower HDL cholesterol than women. They were more frequently smokers and more commonly had coronary artery disease and intermittent claudication compared to women. In contrast, hypertension, heart failure, somatization and CTS were more common among women than among men.

### Atherosclerosis risk factors and CTS

After adjustment for potential confounders, current smoking was associated with possible and probable CTS combined (OR 2.1, 95% 1.4-3.1) and probable CTS (OR 2.9, 95% CI 1.3-6.4). Among current smokers, there was no dose-response relationship between the number of pack-years smoked and CTS (Table 2). BMI, waist circumference and high-sensitive C-reactive protein were associated with operated CTS only. Leisure-time physical activity, alcohol consumption, waist-to-hip ratio, LDL and HDL cholesterol were not associated with CTS. Subjects with type 1 diabetes or metabolic syndrome had a higher prevalence of CTS than those without the condition. Furthermore, the prevalence of operated CTS was higher among subjects with high triglycerides, insulin resistance, or hypertension compared with those without such conditions. None of these associations, however, was statistically significant.

**Table 2:** Adjusted odds ratios (OR) of carpal tunnel syndrome (CTS) according to cardiovascular risk factors.

Characteristic	Sample	Possible and probable CTS combined			Probable CTS			Operated CTS		
				
		Cases	OR	95% CI	Cases	OR	95% CI	Cases	OR	95% CI
Smoking status										
Never smoker	2062	84	1		20	1		29	1	
Former smoker	1649	51	1.2	0.8-1.6	11	1.0	0.4-2.3	15	1.0	0.5-1.8
Occasional smoker	333	9	1.2	0.5-2.6	3	2.0	0.6-6.8	5	2.4	0.9-6.3
Current smoker										
<10 pack-years	501	26	2.0	1.2-3.5	3	1.1	0.3-3.9	6	1.4	0.5-3.7
10-20 pack-years	400	26	2.6	1.5-4.4	12	5.7	2.3-14.1	6	1.8	0.6-4.7
>20 pack-years	429	19	1.7	0.9-3.1	7	2.7	0.9-8.0	6	1.5	0.6-3.7
										
Exercise (times/week)										
≤1	2496	96	1		21	1		29	1	
2-3	1973	68	1.0	0.7-1.5	15	1.0	0.5-1.8	25	1.1	0.6-1.9
≥ 4	1621	69	1.2	0.8-1.6	23	1.6	0.9-3.0	24	1.2	0.6-2.0
										
Alcohol consumption										
None	1237	67	1		19	1		22	1	
Light	972	31	1.0	0.6-1.6	5	0.6	0.2-1.7	9	0.9	0.3-2.2
Moderate	978	25	0.9	0.5-1.5	8	1.1	0.4-2.8	7	0.8	0.3-2.0
Excessive	987	30	1.5	0.8-2.7	6	1.1	0.4-2.9	8	1.3	0.5-3.3
										
Body mass index										
Normal	2514	82	1		18	1		17	1	
Underweight	61	0			0			1	1.9	0.2-15.1
Overweight	2288	82	1.1	0.7-1.5	20	1.1	0.5-2.0	33	2.0	1.1-3.7
Obese	1002	58	1.3	0.8-1.9	19	1.6	0.8-3.0	24	2.8	1.3-5.8
										
Waist circumference										
Normal	2019	59	1		15	1		6	1	
Increased	1684	44	0.8	0.4-1.2	9	0.6	0.2-1.8	20	3.7	1.5-9.0
Obese	2509	137	1.1	0.7-1.6	36	1.1	0.5-2.1	52	4.8	1.9-12.1
										
Waist-to-hip ratio										
Normal	922	24	1		5	1		9	1	
Increased	3554	135	1.3	0.8-2.1	29	1.3	0.5-3.2	38	0.9	0.4-2.2
Obese	1735	81	1.3	0.7-2.2	26	1.6	0.6-4.4	31	1.4	0.5-3.2
										
LDL cholesterol (mg/dL)										
≤129	2072	69	1		23	1		22	1	
130-189	3132	125	1.2	0.8-1.7	26	0.7	0.4-1.2	38	1.1	0.6-1.9
≥190	1032	49	1.4	0.9-2.2	13	1.0	0.5-1.9	19	1.5	0.8-2.9
										
HDL cholesterol (mg/dL)										
≥60	1689	77	1		21	1		22	1	
40-60	3207	119	0.8	0.6-1.2	24	0.7	0.3-1.2	36	0.9	0.5-1.7
≤40	1340	47	0.8	0.5-1.2	17	1.1	0.5-2.2	21	1.5	0.7-2.8
										
Total cholesterol (mg/dL)										
>200	1543	45	1		15	1		12	1	
200-239	2206	91	1.4	0.9-2.3	18	0.8	0.4-1.8	27	1.5	0.7-3.0
≤240	2487	107	1.4	0.9-2.3	29	1.1	0.5-2.2	40	1.8	0.9-3.6
										
Triglycerides (mg/dL)										
≤150	4186	163	1		39	1		46	1	
151-199	1069	42	0.8	0.6-1.1	13	1.0	0.5-1.8	16	1.1	0.5-2.3
≥ 200	981	38	1.0	0.6-1.5	10	1.0	0.4-2.3	17	1.7	0.9-3.1
										
High-sensitive C-reactive protein										
Low (≤3 mg/L)	5117	195	1		46	1		54	1	
High (>3 mg/L)	1062	47	0.9	0.6-1.3	16	1.2	0.6-2.3	25	1.7	1.1-2.8
										
Insulin resistance (tertile)										
1^st^	2077	68	1		18	1		18	1	
2^nd^	2072	83	1.0	0.7-1.4	17	0.8	0.4-1.5	24	1.2	0.7-2.2
3^rd^	2083	92	1.1	0.8-1.6	27	1.1	0.6-1.9	37	1.7	0.9-3.3
										
Metabolic syndrome										
No	4313	145	1		32	1		41	1	
Yes	1902	96	1.1	0.8-1.5	30	1.6	0.98-2.6	36	1.5	0.9-2.5
										
Diabetes										
No	5880	222	1		55	1		71	1	
Type 1	34	2	1.9	0.4-8.5	1	3.5	0.4-29.0	1	-	-
Type 2	319	19	1.0	0.5-1.8	6	1.2	0.4-3.5	7	1.1	0.5-2.6
										
Hypertension										
No	5017	174	1		43	1		52		
Yes	1237	69	1.2	0.8-1.6	19	1.2	0.6-2.1	27	1.6	0.9-2.7

A separate model was run for each characteristic and the odds ratios obtained were adjusted for age, sex, education, somatization, handgrip with high forces and work using vibrating tools

### Atherosclerotic disease and CTS

**Figure 1:**
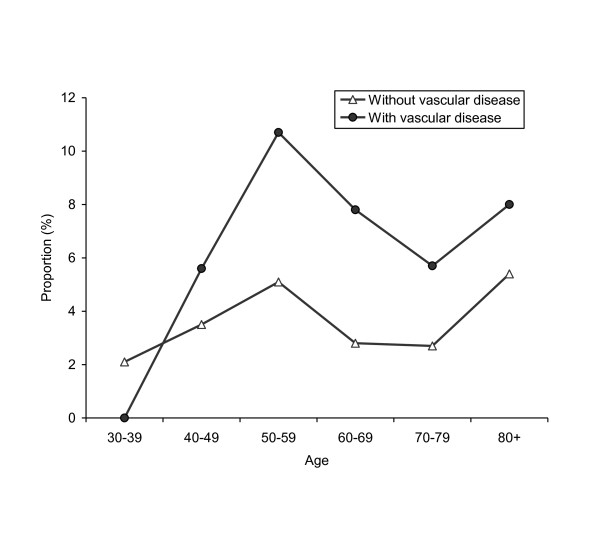
**Age-specific prevalence of possible and probable carpal tunnel syndrome combined according to vascular disease (coronary artery disease, cerebrovascular disease, or intermittent claudication)**. The difference in prevalence between the two groups was statistically significant for the 50-59, 60-69 and 70-79 year age groups.

Subjects with coronary artery disease, valvular heart disease, intermittent claudication, or cerebrovascular disease had a higher prevalence of possible and probable CTS combined (Table 3, Figure 1) or probable CTS (Table 3) compared with those without such a condition. However, none of these associations was statistically significant. Carotid IMT was associated with an increased prevalence of CTS; the odds ratio (OR) was only statistically significant for probable CTS (OR 1.7, 95% CI 1.1-2.6 for each standard deviation (0.23 mm) increase in IMT). Heart failure and arrhythmia were not associated with CTS. Subjects with coronary artery disease were less likely to be operated on for CTS than those without such a condition.

**Table 3:** Adjusted odds ratios (OR) of carpal tunnel syndrome (CTS) according to vascular disease and carotid intima-media thickness.

Cardiovascular disease	Sample	Possible and probable CTS combined			Probable CTS			Operated		
				
		Cases	OR	95% CI	Cases	OR	95% CI	Cases	OR	95% CI
Coronary artery disease										
No	5774	209	1		52	1		75		
Yes	480	34	1.3	0.7-2.2	10	1.5	0.6-3.4	4	0.3	0.1-0.9
										
Intermittent claudication										
No	6175	237	1		60	1		78	1	
Yes	79	6	1.4	0.5-3.6	2	2.1	0.4-9.0	1	0.4	0.1-2.7
										
Cerebrovascular disease										
No	6073	230	1		56	1		78	1	
Yes	181	13	1.2	0.5-2.4	6	2.4	0.8-7.1	1	0.3	0.1-2.3
										
Heart failure										
No	6126	230	1		59	1		77	1	
Yes	128	13	1.2	0.5-2.7	3	1.1	0.3-4.2	2	0.6	0.1-2.6
										
Arrhythmia										
No	5916	221	1		56	1		75	1	
Yes	338	22	1.0	0.6-1.8	6	1.2	0.4-3.4	4	0.6	0.2-1.9
										
Valvular heart disease										
No	6078	229	1		57	1		77	1	
Yes	176	14	1.6	0.7-3.4	5	2.3	0.7-6.4	2	0.7	0.1-3.2
										
Carotid intima-media thickness										
Mean IMT, per each standard deviation (0.23 mm) increase	1353	55	1.3	0.99-1.8	14	1.7	1.1-2.6	16	1.4	0.9-2.2

A separate model was run for each characteristic and the odds ratios obtained were adjusted for age, sex, education, somatization, handgrip with high forces and work using vibrating tools

### Age-specific analyses

#### Possible and probable CTS combined

BMI, waist-to-hip ratio, LDL cholesterol, triglycerides, hypertension, and arrhythmia were associated with possible and probable CTS combined in subjects aged 30-44 (Table 4). In the age group of 60 years or over, leisure-time physical activity, coronary artery disease, and valvular heart disease were associated with CTS. The association between current smoking and CTS was only statistically significant in subjects aged 45-59. Alcohol consumption, C-reactive protein, metabolic syndrome, and insulin resistance were not associated with CTS in any age group.

**Table 4:** Age-specific adjusted odds ratio of possible and probable carpal tunnel syndrome combined, Health 2000 survey, 2000-2001.

Characteristic	30-44 yrs (N = 2132)				45-59 yrs (N = 2190)				60+ yrs (N = 1932)			
			
	Sample	Cases	OR	95% CI	Sample	Cases	OR	95% CI	Sample	Cases	OR	95% CI
Smoking status												
Never smoker	522	9	1		593	29	1		947	46	1	
Former smoker	465	8	0.9	0.3-2.4	649	23	1.0	0.6-1.7	535	20	1.6	0.9-3.0
Occasional smoker	194	3	0.8	0.2-3.2	105	6	1.8	0.6-4.8	34	0	-	
Current smoker	601	22	1.8	0.8-3.6	553	42	1.9	1.1-3.3	193	8	1.8	0.7-4.4
												
Exercise (times/week)												
≤1	915	23	1		916	45	1		665	28	1	
2-3	775	16	0.8	0.4-1.6	717	35	1.0	0.6-1.7	481	17	1.2	0.6-2.4
≥ 4	427	9	0.9	0.4-1.8	526	27	1.1	0.6-1.8	668	33	1.7	1.0-2.7
												
Body mass index												
Normal	1065	18	1		815	34	1		634	30	1	
Overweight	688	15	1.4	0.7-2.7	848	43	1.1	0.6-1.8	752	24	0.7	0.4-1.3
Obese	264	14	2.4	1.1-5.4	413	27	1.2	0.6-2.2	325	17	0.7	0.3-1.5
												
Waist circumference												
Normal	1015	18	1		617	26	1		387	15	1	
Increased	562	9	0.8	0.3-2.2	614	22	0.7	0.4-1.3	508	13	0.6	0.2-1.2
Obese	535	22	1.5	0.8-2.9	954	62	0.9	0.5-1.6	1020	53	0.8	0.4-1.5
												
Waist-hip ratio												
Normal	484	4	1		281	11	1		157	9	1	
Increased	1271	32	2.7	0.9-7.8	1202	64	1.5	0.7-2.9	1081	39	0.5	0.2-1.2
Obese	357	13	3.0	1.0-9.0	702	35	1.1	0.5-2.3	676	33	0.7	0.3-1.5
												
LDL cholesterol (mg/dL)												
<129	951	16	1		604	28	1		517	25	1	
130-189	973	23	1.6	0.8-3.2	1163	62	1.2	0.7-2.0	996	40	0.8	0.4-1.4
≥190	198	10	3.8	1.6-9.1	419	20	1.1	0.5-2.3	415	19	0.8	0.4-1.6
												
HDL cholesterol (mg/dL)												
>60	590	15	1		609	41	1		490	21	1	
40-60	1121	22	0.7	0.3-1.4	1129	52	0.7	0.4-1.2	957	45	1.2	0.6-2.1
≤40	411	12	1.3	0.6-2.9	448	17	0.6	0.3-1.0	481	18	1.0	0.4-2.2
												
Total cholesterol (mg/dL)												
>200	759	13	1		393	18	1		391	14	1	
200-239	797	16	1.1	0.4-2.5	785	44	1.3	0.6-2.5	624	31	1.6	0.7-3.4
≤240	566	20	2.3	0.97-5.5	1008	48	1.1	0.5-2.2	913	39	1.2	0.5-2.3
												
Triglycerides (mg/dL)												
≤150	1562	32	1		1435	78	1		1189	53	1	
151-199	281	4	0.6	0.2-1.8	395	21	0.8	0.5-1.3	393	17	0.7	0.4-1.3
>200	279	13	2.7	1.2-6.1	356	11	0.6	0.3-1.1	346	14	0.9	0.5-1.9
												
Hypertension												
No	2028	41			1748	83			1241	50		
Yes	104	8	3.4	1.6-7.4	442	27	1.0	0.6-1.7	691	34	1.0	0.6-1.5
												
Coronary artery disease												
No	2127	49			2141	107			1506	53		
Yes	5	0	-	-	49	3	1.2	0.3-3.8	426	31	1.9	1.1-3.5
												
Heart failure												
No	2131	49			2184	109	1		1811	72	1	
Yes	1	0	-	-	6	1	2.0	0.2-15.0	121	12	1.6	0.6-3.8
												
Arrhythmia												
No	2118	47	1		2118	103	1		1680	71	1	
Yes	14	2	10.2	2.7-38.4	72	7	1.6	0.6-3.8	252	13	0.8	0.4-1.8
												
Valvular heart disease												
No	2122	49			2161	108			1795	72		
Yes	10	0	-	-	29	2	1.0	0.2-4.8	137	12	2.3	1.0-5.0
												
Mean IMT, per each standard deviation (0.23 mm) increase					876	38	1.2	0.8-1.9	477	17	1.4	0.9-2.1

A separate model was run for each characteristic and the odds ratios obtained were adjusted for age, sex, education, somatization, handgrip with high forces and work using vibrating tools

#### Probable CTS

The results remained consistent using probable CTS as outcome. The prevalence of probable CTS increased two-fold (OR 2.1, 95% CI 1.4-3.1) for each standard deviation increase in IMT for subjects aged 60-74. The association was weak and non-significant among those aged 45-59 (OR 1.4, 95% CI 0.9-2.1). The prevalence of probable CTS was higher among subjects with vascular disease (coronary artery disease, cerebrovascular disease or intermittent claudication) aged 50 or over, and among subjects with hypertension aged 30-39 or over 70, compared with those without such a condition (data not shown).

#### Operated CTS

Metabolic syndrome, C-reactive protein, LDL and HDL cholesterol, and insulin resistance were associated with operated CTS only in subjects aged 45-59 (data not shown). BMI was associated with CTS among subjects aged 30-44 as well as among those aged 45-59. Former smoking and physical activity were associated with operated CTS among those aged 60 or over. Among subjects with hypertension, operated CTS had a bimodal age distribution with a first peak in those aged 50-59 and a second peak in those aged 70-79.

### Gender-specific analyses

In gender-specific analyses, the associations of atherosclerosis risk factors with possible and probable CTS combined did not differ between men and women. After adjustment for potential confounders, carotid IMT was associated with possible and probable CTS combined (OR 1.7, 95% CI 1.1.2.6 for each 0.23 mm increase), probable CTS (OR 2.2, 95% CI 1.2-4.0), and operated CTS (OR 2.3, 95% CI 1.1-4.8) among men only.

### Effect modification

In subjects aged 30-44, stratified analyses controlled for potential confounders showed that high LDL cholesterol, high triglycerides, hypertension, and insulin resistance were associated with CTS in overweight or obese subjects, but not in normal-weight subjects (Table 5). Mean BMI did not differ in overweight/obese subjects with an LDL of >130 vs. ≤129 (28.9 vs. 28.6 kg/m^2^). It was higher in overweight/obese subjects with triglycerides of >150 vs. <150 (29.6 vs. 28.2 kg/m^2^), overweight/obese subjects with vs. without hypertension (30.9 vs. 28.6 kg/m^2^), and in overweight/obese subjects with high vs. low insulin resistance (29.7 vs. 27.4 kg/m^2^). Among subjects aged 60 or over, the association of vascular disease with CTS was independent of BMI level.

**Table 5:** Odds ratio of possible and probable carpal tunnel syndrome combined for joint effects of body mass index and serum lipids or hypertension in subjects aged 30-44 (N = 2132)

Characteristic	Sample	Cases	OR	95% CI
BMI (kg/m^2^) and LDL cholesterol (mg/dL)				
BMI <25 and LDL cholesterol <129	550	8	1	
BMI ≥25 and LDL cholesterol <129	355	8	1.5	0.5-4.1
BMI <25 and LDL cholesterol ≥130	532	10	1.2	0.4-3.3
BMI ≥25 and LDL cholesterol ≥130	594	21	2.7	1.2-6.4
				
BMI (kg/m^2^) and triglycerides (mg/dL)				
BMI <25 and triglycerides ≤150	945	16	1	
BMI ≥25 and triglycerides ≤150	550	14	1.5	0.7-3.4
BMI <25 and triglycerides >150	137	2	0.9	0.2-4.7
BMI ≥25 and triglycerides >150	399	15	2.9	1.3-6.4
				
BMI (kg/m^2^) and blood pressure				
BMI <25 and normal blood pressure	1062	17	1	
BMI ≥25 and normal blood pressure	881	23	1.8	0.9-3.4
BMI <25 and high blood pressure	27	1	2.2	0.2-19.0
BMI ≥25 and high blood pressure	71	6	5.9	2.1-16.3
				
BMI (kg/m^2^) and insulin resistance *				
BMI <25 and low insulin resistance	1819	55	1	
BMI ≥25 and low insulin resistance	1057	42	1.5	0.6-3.2
BMI <25 and high insulin resistance	745	27	0.6	0.2-2.0
BMI ≥25 and high insulin resistance	2225	98	2.1	1.1-4.0

* Insulin resistance (serum insulin × glucose/22.5) was dichotomized using the median

Odds ratios are adjusted for age, sex, education, handgrip with high forces and work using vibrating tools

After adjustment for age, gender and education, carotid IMT was only associated with possible and probable CTS combined in subjects with hypertension (OR 1.6, 95% CI 1.1-2.4 for each 0.23 mm increase in IMT), and not in those with normal blood pressure. This association did not differ between men (OR 1.6, 95% CI 0.9-2.6) and women (OR 1.6, 95% CI 0.8-2.8). Furthermore, carotid IMT was associated with possible and probable CTS combined only in subjects with vascular disease (coronary artery disease, intermittent claudication or cerebrovascular disease) (OR 2.1, 95% CI 1.1-4.0). Finally, carotid IMT was associated with CTS only among subjects who were exposed to physical workload factors (Table 6). The odds ratio was 1.5 (95% CI 1.0 -2.2) for subjects exposed to handgrip with high forces, 2.1 (95% CI 1.2-3.7) for those exposed to manual handling of loads over 20 kg, and 2.6 (95% CI 1.4-4.8) for subjects exposed to both high handgrip forces and manual handling of loads over 20 kg.

**Table 6:** Odds ratio of possible and probable carpal tunnel syndrome combined for each standard deviation (0.23 mm) increase in mean carotid intima-media thickness according to exposure to physical workload factors in subjects aged 45-74.

Physical workload factor	Sample	Cases	OR *	95% CI
Handgrip with high forces				
No	979	26	1.1	0.6-1.7
Yes	363	29	1.5	1.0-2.2
				
Manual handling of loads >20 kg				
No	1091	39	1.1	0.7-1.6
Yes	253	16	2.1	1.2-3.7
				
Use of vibrating tools				
No	1233	49	1.3	0.9-1.8
Yes	111	6	1.4	0.7-2.5
				
Handgrip with high forces and handling of loads >20 kg				
None	908	25	1.1	0.7-1.8
Only handgrip with high forces	182	14	1.1	0.5-2.0
Only handling of loads >20 kg	71	1	-	-
Both	181	15	2.6	1.4-4.8

* Adjustment for age, gender and education

There was no interaction between smoking and obesity, between smoking and physical workload factors, and between obesity and physical workload factors.

## Discussion

Our findings showed that obesity, dyslipidemia, and hypertension are associated with CTS in people aged 30-44, while coronary artery disease and carotid IMT are associated with CTS in those aged 60 or over. An association between carotid IMT and CTS was found only in subjects exposed to physical load factors and in those with hypertension or atherosclerotic disease. Our study [10] is in line with other studies [4,5] showing a bimodal age distribution for CTS among men [4,5] and women [4], which may partly be due to atherosclerosis and its risk factors.

The aetiology of CTS is multifactorial. Some studies have shown associations between cardiovascular risk factors such as obesity, smoking, LDL cholesterol and metabolic syndrome, and CTS [16,17,23-27]. We found associations of atherosclerosis risk factors with CTS in young adults. Other studies [24,34] have also reported a stronger association of obesity with CTS in younger than older subjects. Overweight/obesity with hypertension or metabolic disturbances such as dyslipidemia and insulin resistance may play a role in CTS among young adults. Subjects with hypertension or dyslipidemia are more likely to have a higher BMI than those with normal blood pressure or normal serum lipid levels. Moreover, for women, hormonal factors may also play a role in CTS [8]. Therefore, the associations of hypertension, LDL cholesterol and triglycerides with CTS could be confounded by BMI, oral contraceptive use, or hormone replacement therapy. However, in the current study, after further adjustment for BMI (both in total population and in women) and history of oral contraceptive use or hormone replacement therapy (in women), these associations remained statistically significant in subjects aged 30-44 (data not shown). The use of oral contraceptives only slightly attenuated the association between triglycerides and CTS.

So far, there is no strong evidence to support an association between atherosclerosis and CTS. Carotid artery IMT is used as a surrogate marker of early atherosclerosis or a measure of asymptomatic atherosclerotic vascular disease [35]. We found a stronger association of carotid IMT with CTS in older than younger subjects. This may be due to the fact that atherosclerosis was more advanced among subjects aged 60-74 (Mean IMT 1.06, SD 0.25) than among those aged 45-59 (Mean IMT 0.87, SD 0.18). Moreover, there was an association between carotid IMT and CTS only in subjects with atherosclerotic vascular disease or in those exposed to physical load factors. In subjects with vascular disease, carotid IMT may also be associated with obliterative changes in arteries supplying the carpal tunnel contents. Impairment of vascular supply could render the median nerve more vulnerable to mechanical loads.

Ischemic endothelial damage leads to increased vascular permeability and increased interstitial fluid pressure [11,12]. This may render the median nerve vulnerable to damage especially when the oxygen demands of the carpal tunnel contents due to physical stress are increased. The ischemia of the median nerve produces neuronal edema and subsequently leads to intraneural fibrosis. The association between carotid IMT and CTS in those suffering from vascular disease may be related to the severity of such a disease. In other analyses, we found that subjects with a thicker carotid intima-media have several coexisting vascular diseases or risk factors (data not shown).

The advantages of the current study include a population-based sample with a high response rate, face-to-face interviews, comprehensive physical examinations, laboratory tests, and the advanced imaging method. The study population was interviewed on two separate occasions and by different interviewers in order to gather information on both exposure history and musculoskeletal symptoms. Therefore, those who assessed exposures were not aware of musculoskeletal symptoms [28]. The limitations of this study are its cross-sectional nature and its reliance on CTS diagnosed primarily by physical examination. We did not use nerve conduction studies to confirm the diagnosis of CTS. Considering nerve conduction studies as a golden standard test, a combination of a classic/probable hand diagram and either a positive Tinel's or Phalen's test result can correctly diagnose 79% of CTS cases [36]. Our prevalence estimate (3.8%) was also close to those obtained in a Swedish population-based study [1] through a clinical examination (3.8%) or electrodiagnostic measurements (4.9%).

The results of subgroup analyses should be interpreted cautiously, as the sample size was relatively small. Some of the observed associations, however, are less likely to be due to chance. Most of the common atherosclerosis risk factors were associated with CTS among young subjects. Moreover, after correcting for multiple testing (Bonferroni correction), the associations of high LDL cholesterol, hypertension and cardiac arrhythmia with CTS remained statistically significant in subjects aged 30-44. In those aged 45-74, the joint effect of forceful activities and thick carotid intima-media remained statistically significant. Furthermore, other comorbid conditions such as cancer, psychosis, osteoporosis, allergy, dementia and a chronic illness or inflammation of the bowel were not associated with CTS.

## Conclusions

The current study may support the role of atherosclerosis and its risk factors in the aetiology of CTS. Of the two peaks in the age-specific prevalence of CTS, the first, occurring after the age of 40, may largely be due to work-related factors and the risk factors of atherosclerosis. The second peak in those aged 70 or over [4,5,10], may be explained by ischemic vascular disease and atherosclerosis. Our findings suggest that CTS may be a manifestation of atherosclerosis, or that both conditions may have common risk factors. Therefore, effective population-level health promotion activities against cardiovascular risk factors may reduce the risk not only of atherosclerosis and vascular diseases, but also of CTS.

## Conflict of interests

The authors declare that they have no competing interests.

## Authors' contributions

MH, LM and EV-J participated in the design of the study and its analysis. RS carried out the statistical analyses and drafted the manuscript. All authors critically revised the manuscript and approved the final version to be submitted for publication.

## Pre-publication history

The pre-publication history for this paper can be accessed here:

http://www.biomedcentral.com/1471-2474/12/80/prepub
